# Dexamethasone Ameliorates H_2_S-Induced Acute Lung Injury by Alleviating Matrix Metalloproteinase-2 and -9 Expression

**DOI:** 10.1371/journal.pone.0094701

**Published:** 2014-04-10

**Authors:** Jun Wang, Huazhong Zhang, Chenglei Su, Junjie Chen, Baoli Zhu, Hengdong Zhang, Hang Xiao, Jinsong Zhang

**Affiliations:** 1 Department of Emergency Medicine, the First Affiliated Hospital of Nanjing Medical University, Nanjing, Jiangsu, China; 2 Key Lab of Modern Toxicology, Ministry of Education, Department of Toxicology, School of Public Health, Nanjing Medical University, Nanjing, Jiangsu, China; 3 Department of Occupational Disease Prophylactico-Therapetic Institution, Jiangsu Provincial Center for Disease Prevention and Control, Nanjing, Jiangsu, China; Chang Gung University, Taiwan

## Abstract

Acute lung injury (ALI) is one of the fatal outcomes after exposure to high levels of hydrogen sulfide (H_2_S), and the matrix metalloproteinases (MMPs) especially MMP-2 and MMP-9 are believed to be involved in the development of ALI by degrading the extracellular matrix (ECM) of blood-air barrier. However, the roles of MMP-2 and MMP-9 in H_2_S-induced ALI and the mechanisms of dexamethasone (DXM) in treating ALI in clinical practice are still largely unknown. The present work was aimed to investigate the roles of MMP-2 and MMP-9 in H_2_S-induced ALI and the protective effects of DXM. In our study, SD rats were exposed to H_2_S to establish the ALI model and in parallel, A549 cells were incubated with NaHS (a H_2_S donor) to establish cell model. The lung HE staining, immunohistochemisty, electron microscope assay and wet/dry ratio were used to identify the ALI induced by H_2_S, then the MMP-2 and MMP-9 expression in both rats and A549 cells were detected. Our results revealed that MMP-2 and MMP-9 were obviously increased in both mRNA and protein level after H_2_S exposure, and they could be inhibited by MMP inhibitor doxycycline (DOX) in rat model. Moreover, DXM significantly ameliorated the symptoms of H_2_S-induced ALI including alveolar edema, infiltration of inflammatory cells and the protein leakage in BAFL via up-regulating glucocorticoid receptor(GR) to mediate the suppression of MMP-2 and MMP-9. Furthermore, the protective effects of DXM in vivo and vitro study could be partially blocked by co-treated with GR antagonist mifepristone (MIF). Our results, taken together, demonstrated that MMP-2 and MMP-9 were involved in the development of H_2_S-induced ALI and DXM exerted protective effects by alleviating the expression of MMP-2 and MMP-9. Therefore, MMP-2 and MMP-9 might represent novel pharmacological targets for the treatment of H_2_S and other hazard gases induced ALI.

## Introduction

Hydrogen sulfide (H_2_S), a colorless gas with a characteristic rotten-egg odor, is associated with more than 70 types of industries, including petroleum refineries, paper and pulp manufacture, sewage treatment, and artificial fiber synthesis [1]. The primary mechanism for the toxic effects of H_2_S is direct inhibition of cytochrome oxidase system, thereby arresting aerobic cellular respiration [2]. Inhaling H_2_S associated with hazardous material accidents in industrial situation resulted in fatal outcomes were previously reported [3]–[5]. And the toxic effects of H_2_S depend on its concentration and the duration of exposure. It is immediately fatal when concentrations are 500–1,000 ppm, while exposed to lower concentrations(10–500 ppm), various respiratory symptoms that range from rhinitis to acute lung injury(ALI)/acute respiratory distress syndrome(ARDS) may occur, of all, the respiratory failure of ALI/ARDS has been largely attributed to death in H_2_S poisoning [6]–[8].

In the pathogenesis of ALI/ARDS, disruption of the alveolar epithelial-endothelial capillary barrier(also known as the blood-air barrier), which consists of alveolar epithelium, capillary endothelium, extracellular matrix (ECM), alveolar macrophages and other cells is considered as a central event [9], [10]. The matrix metalloproteinases (MMPs) are believed to be the main physiological mediators of ECM degradation, under normal conditions, MMPs are secreted from cells as inactive forms(pro-MMPs), however, most of MMPs can be activated and significantly secreted during the systemic inflammation response and tissue damage, such as ALI/ARDS, which was characterized by the disruption of blood-air barrier. In MMPs family, MMP-2 (gelatinase A, pro-MMP-2 72 kDa, active-MMP-2 62 kDa) is synthesized by a wide variety of cells including alveolar epithelial cells(AECs), endothelial cells and fibroblasts. MMP-9 (gelatinase B, pro-MMP-9 92 kDa, active-MMP-9 82 kDa) is produced mainly by inflammatory cells like polymorphonuclear neutrophils(PMNs) and macrophages. Both MMP-2 and MMP-9 were previously reported to degrade basement membrane(BM) the major structure of ECM [11], [12]. Reports implicating MMP-2 and MMP-9 in various models of ALI/ARDS have been found in a spectrum of literatures [13]–[16], and the MMPs knockout mouse showed less severe lung injury than the wide type in immune complex-induced ALI [17], [18]. However, the roles of MMP-2 and MMP-9 in H_2_S-induced ALI are still unknown.

Dexamethasone(DXM), one of the glucocorticoids, is widely used in clinical practice for many years with strong anti-inflammatory actions, it routinely administered for many respiratory diseases, including ALI/ARDS [19]. Recent reports proved that glucocorticoid receptor(GR) was involved in the pathogenesis of ALI/ARDS and GR would be the key target in the ALI/ARDS treatment [20], [21]. In addition to the strong anti-inflammatory effects of glucocorticoids, available studies have also shown that GR mediate the suppression of MMPs, and glucocorticoids could inhibit MMPs expression in the cases of injury [22]–[25]. Therefore, the MMP inhibitor DOX was used in the present study to investigate the roles of MMP-2 and MMP-9 after H_2_S exposure, and evaluate the therapeutic effects of DXM on H_2_S-induced ALI. In vivo study, SD rats were exposed to H_2_S to establish ALI model and in parallel, for the in vitro experiment, A549 cells, which commonly used as a model of AECs [26], were incubated with NaHS(a widely used H_2_S donor) to establish cell model [27], [28]. Our study, for the first time, clarified the effects of MMP-2 and MMP-9 in H_2_S-induced ALI, and also suggested that DXM might exert its protective effects through down-regulation of MMP-2 and MMP-9 expression.

## Material and Methods

### 1. Materials

NaHS, doxycycline, dexamethasone, mifepristone (RU486), Bovine albumin and sodium pentobarbital were purchased from Sigma (St. Louis, MO, USA). Rabbit anti-MMP-2 antibody and rabbit anti-MMP-9 antibody were obtained from Millipore (Bedford, MA, USA). Goat anti-GAPDH antibody was obtained from GenScript (Piscataway, NJ, USA). Gas cylinders containing 1% (10,000 ppm) H_2_S standardgases was purchased from ShangYuan GASES (Nanjing, China). A digital H_2_S gas analyzer was purchased from Lasting Star Safety Equipment Company (Nanjing, China). All other reagents were all from Sigma if not otherwise specially stated.

### 2. Animals and cells

Male Sprague-Dawley rats weighing 200–250 g were approved by Animal Center of Jiangsu Province, Nanjing, China (SCXK (Su) 2002-0031) with free access to standard rat chow and tap water. Animal rooms were ventilated with HEPA-filtered air and maintained at 18.5–21.5°C and 40–70% humidity on a 12-h light-dark cycle. All animal protocols were approved by the Institutional Animal Care and Use Committee (IACUC) of Nanjing Medical University (China) (Permit Number: 20110521).

A549 alveolar epithelial cell line seeded at 4×10^5^cells/cm^2^ in 25 cm^2^ flasks (Corning) were obtained from American Type Culture Collection (ATCC). A549 cells were maintained in RPMI 1640 medium (Hyclone) with 10% fetal bovine serum (FBS, Hyclone) and 1% penicillin/streptomycin at 37°C in a humidified incubator in 5% CO2 atmosphere.

### 3. H_2_S exposure

#### 3.1 Rat model

Methods used to generate and characterize the H_2_S exposure atmospheres are according to previously described [1], [29]. Briefly, two mass flow controllers sealed with Kalrez and a digital H_2_S gas analyzer were used to blend air and H_2_S mixtures to the target H_2_S concentrations in a custom-built 0.7 m^3^ sealed plexiglas exposure chamber. Rats were acute exposed to sublethal concentrations of inhaled H_2_S(300 ppm) for 3 h in the chamber [30], [31], then returned to room air for the subsequent study.

#### 3.2 Cell model

NaHS, a H_2_S donor, was dissolved in PBS at 100 mM for the stocking concentration. The A549 cells were kept in RPMI 1640 medium (without FBS) prior to NaHS treatment. The diluted NaHS (500 μM) was used to incubate with A549 cells for 6 h, 12 h and 24 h. Since hydrogen sulfide can escape as a gas from the solutions, the flasks were sealed for 30 min in order to establish the homogeneous and reproducible toxic results [27], [32].

### 4. Experimental design

The first aim of the present work was to evaluate the expression of MMP-2 and MMP-9 in lung tissues and A549 cells following H_2_S exposure. In vitro study, A549 cells were incubated with NaHS for 6 h, 12 h and 24 h, while in vivo study, 35 rats were randomly divided into control(unexposed) group and six time point groups (n = 5 per group) using a computer-generated randomization schedule. Control rats were kept in room air, others 30 rats were exposed to 300 ppm H_2_S for 3 h, then they were returned to room air and anesthetized by intraperitoneal administration sodium pentobarbital to remove lung tissues at 30 min, 1 h, 3 h, 6 h, 12 h and 24 h after H_2_S exposure. After that, the effects of H_2_S exposure on MMP-2 and MMP-9 expression were detected both in vivo and in vitro by realtime-PCR and western-blot.

Our second objective was to further confirm the roles of MMP-2 and MMP-9 in H_2_S-induced ALI with DOX by inhibition of MMP-2 and MMP-9. 34 rats were randomly divided into five groups(n = 5/8 per group) as described in Table 1, DOX treatment was administered daily by gavage with DOX(20 mg/kg in 2 ml water) for consecutive 7 days [33], [34], then rats were exposed to 300 ppm H_2_S for 3 h, 9 rats(n = 3 per group) were anesthetized to execute the bronchoalveolar lavage(BAL) at the time point of 6 h after exposure, and other 25 rats were killed 1 h or 6 h later to get tissue specimens for further use.

**Table 1:** Groups of DOX.

Group	n	Application
Control group	8	tissue specimen and BAL
H_2_S post 1 h group	5	tissue specimen
H_2_S post 6 h group	8	tissue specimen and BAL
DOX pretreated + H_2_S post 1 h group	5	tissue specimen
DOX pretreated + H_2_S post 6 h group	8	tissue specimen and BAL

We were also interested in determining whether DXM could ameliorate H_2_S-induced ALI by alleviating MMP-2 and MMP-9 expression. In order to verify our hypothesis, 71 rats were randomly divided into ten groups(n = 5/8 per group) as described in Table 2, DXM(2 mg/kg/day) and/or glucocorticoid receptor antagonist mifepristone (MIF,10 mg/kg/q12 h) diluted in corn oil were intraperitoneal injection for consecutive 3 days prior to H_2_S exposure [35]–[38]. On the 3rnd day, rats were exposed to 300 ppm H_2_S for 3 h, then returned to room air, 21 rats(n = 3 per group) were anesthetized to execute the BAL, and others were killed 1 h or 6 h later to get tissue specimens. In A549 cells, DXM and MIF diluted in ethanol and stored at 10 mM. A549 cells were kept in RPMI 1640 medium without FBS and co-incubated with DXM (100 nM) and/or MIF (1 μM) for 24 hours [39], then NaHS (500 μM) was added to incubated with A549 cells for 6 h and 12 h, then MMP-2 and MMP-9 expression were detected.

**Table 2:** Groups of DXM and MIF.

Group	n	Application
Control group	8	tissue specimen and BAL
H_2_S post 1 h group	5	tissue specimen
H_2_S post 6 h group	8	tissue specimen and BAL
DXM pretreated + H_2_S post 1 h group	5	tissue specimen
DXM pretreated + H_2_S post 6 h group	8	tissue specimen and BAL
DXM and MIF pretreated +H_2_S post 1 h	5	tissue specimen
DXM and MIF pretreated +H_2_S post 6 h	8	tissue specimen and BAL
DXM treated control(unexposed) group	8	tissue specimen and BAL
MIF treated control(unexposed) group	8	tissue specimen and BAL
DXM and MIF treated control(unexposed) group	8	tissue specimen and BAL

### 5. Lung wet-to-dry weight ratio and BALF analyses

To evaluate the severity of pulmonary edema, the lung wet-to-dry weight ratio was detected [40]. The upper lobe of the right lung was removed and weighted. Then specimens were drying in an oven (50°C)for 3 days and weighed again to determine the dry weight. The wet/dry ratio was calculated by dividing the wet weight by the dry weight. The BAL was performed using an endotracheal tube inserted into the trachea at 6 h after H_2_S exposure. Three aliquots of 3 mL 0.9% sterile NaCl were administered through a three way tube at 15 cm H_2_O pressure by gravity, sustained in lung for 3 minutes, then aspirated by suction [41]. Generally, the BAL fluid return was approximately half of the instilled fluid. No other samples were collected from these animals. The protein content in bronchoalveolar lavage fluid (BALF) was determined by the Pierce BCA Protein Assay (Thermo Scientific, USA) using a spectrophotometer (Beckman Coulter, Los Angeles, CA, USA) at a wavelength of 564 nm.

### 6. Lung histology evaluation

#### 6.1 H&E stain

For light microscope analysis, the right lower lobe from each rat was harvested and fixed in 4% paraformaldehyde for 24 hours, then embedded in paraffin and cut into 5 μm-thick serial sections, finally stained with hematoxylin and eosin (H&E). Pathologic changes were evaluated by two independent observers, who had no knowledge of the H_2_S exposure. The following four parameters: congestion and edema, hemorrhage, inflammatory cells, and septal thickening, which manifested the characteristics of ALI were assessed. Then they graded on a four-point scale(0 = absent, 1 = mild, 2 = moderate, 3 = severe), an overall histological score was calculated by totalling the scores as previously described [42].

#### 6.2 Immunohistochemical Examination

For immunohistochemical examination, 3 μm-thick serial sections from paraffin-embedded tissue, were prepared with deparaffinizing, rehydrating and quenching endogenous peroxidase. Then sections were microwaved in 10 mM sodium citrate buffer (pH 6.0) for antigen retrieval. Each section was incubated with rabbit monoclonal MMP-2 or MMP-9 (1: 100, Epitomics, USA) for 1 hour at room temperature and overnight at 4°C. Following the reaction with anti-rabbit IgG (1∶50000, Jackson, USA) for 15 minutes, the sections were treated with aminoethyl carbazole and counterstained with Mayer's hematoxylin. Images of H&E stain and immunohistochemical examination were both processed by a Nikon eclipse 80i microscope with NIS Elements software (Media Cybernetics, Silver Spring, USA).

#### 6.3 Transmission Electron Microscope

For ultra-structural studies, two parts of the lungs were collected, one from the cranial and another from the caudal aspect of the lateral portion of the left lung, were processed and embedded in Spurrresin. One-pm-thick sections were cut and stained with toluidine blue. Thin sections of preselected areas of the bronchoalveolar region and vasculature were cut and stained with uranyl acetate and lead citrate. The images were taken by transmission electron microscope (Philips, TEM-400).

### 7. RNA extraction and Real-time PCR (Q-PCR) analysis

Total RNA was isolated using RNAiso Plus (TaKaRa, Japan) according to the manufacturer's instructions. RNA was dissolved in RNase-free water and concentrations were assessed by NANO drop ND-1000 Spectrophotometer (Nano Drop Technologies). Then mRNA reverse transcribed into cDNA using RT-PCR kit (TaKaRa, Japan). The primer sequences were listed in Table 3. Real-time PCR was carried out on the ABI Prism 7300 HT Sequence Detection System with SYBR Premix Ex Taq kit (TaKaRa, Japan) in 20 μL reaction mixture. Fold changes in mRNA levels were calculated by using the ΔΔCT method and GAPDH as reference gene.

**Table 3:** The primer sequences.

Product	Sequence		Length, bp	Acc.No
**Primers used for rat lung**				
MMP-2	sense	CCCCTATCTACACCTACACCAA	194	NM_031054.2
	antisense	CTCACCACGGATCTGAGCAAT		
MMP-9	sense	AAAGGTCGCTCGGATGGTTAT	159	NM_031055.1
	antisense	CTGCTTGCCCAGGAAGACGAA		
GAPDH	sense	ACATCATCCCTGCATCCACT	258	NM_017008.4
	antisense	GGGAGTTGCTGTTGAAGTCA		
**Primers used for A549 cells**				
MMP-2	sense	TGTGTTCTTTGCAGGGAATGAAT	145	NM_001127891.1
	antisense	TGTCTTCTTGTTTTTGCTCCAGTTA		
MMP-9	sense	CCTCTGGAGGTTCGACGTGA	123	NM_004994.2
	antisense	TAGGCTTTCTCTCGGTACTGGAA		
GAPDH	sense	CGCTGAGTACGTCGTGGAGTC	172	NM_001256799.2
	antisense	GCTGATGATCTTGAGGCTGTTGTC		

### 8. Western-blot analysis

Sample proteins were prepared by using the RIPA (Sigma, USA) buffer with Protease Inhibitor Cocktail (Sigma, USA) and then incubated for 30 min at 4°C. The protein concentration was measured with Pierce BCA Protein Assay (Thermo Scientific, USA). An equal amount of protein (20 μg) was loaded onto Tris-glycine sodium dodecylsulphate (SDS) polyacrylamide gel (10%) for electrophoresis, and subsequently blotted onto a PVDF (Millipore, USA) membrane. The membranes were blocked with 5% nonfat milk in TBST for 2 hours, and then incubated with anti-MMP-2 (dilution 1∶1,000), anti-MMP-9 (1∶1,000), goat anti-GAPDH (1∶4,000) for at least 8 hours. After incubated with HRP-conjugated anti-rabbit or anti-goat secondary antibody (1∶50000, Jackson ImmunoResearch Laboratories, USA) for 1 h at room temperature. Labeled proteins were visualized by Pierce ECL Western Blotting Substrate (Thermo Scientific, USA). The relative content of target proteins were detected by Molecular Imager Gel Doc XR + System with Image-Lab software (Bio-Rad,USA). Band density was normalized to GAPDH in each sample.

### 9. Statistical analysis

Data are expressed as mean±S.E. for all the experiments. Statistically significant differences between the treatments and the control were determined by one-way ANOVA or the Student's t-test. All tests of statistical significance were two-sided and the statistical significance was set at P<0.05.

## Results

### 1. Assessment of ALI after H_2_S exposure in rats

**Figure 1:**
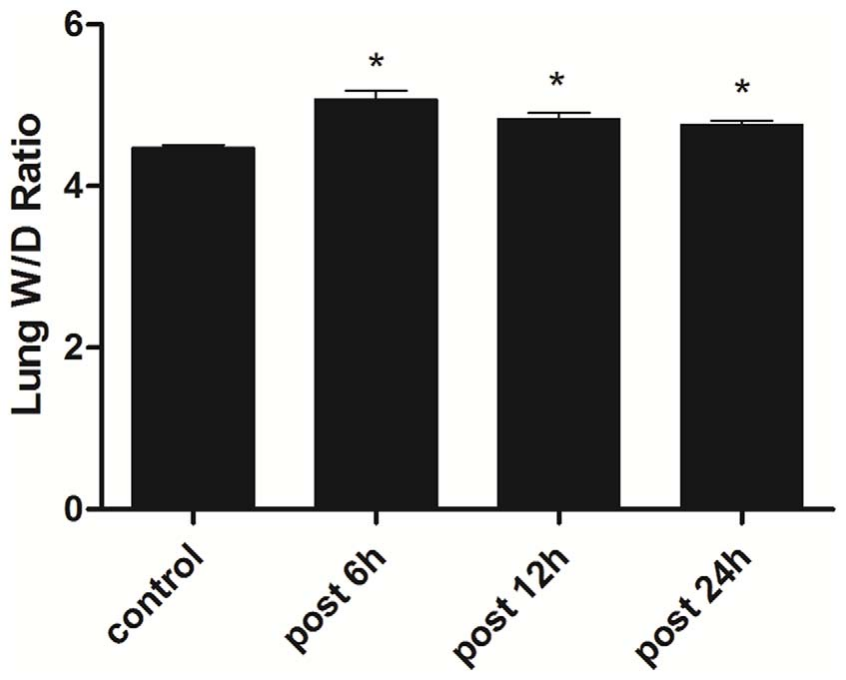
Effects of H_2_S on the lung W/D ratio. 6_2_S, and the W/D ratio was detected. * Indicates significant difference (p<0.05) when the values were compared to the control.

Dysphoria and polypnea were observed after 15 min when rats exposed to H_2_S(300 ppm), 1 h later the mucous membrane irritation occured, including nasal congestion, eye conjunctival congestion edema with aggravated difficulty in breathing as previously described [1], [43]. After H_2_S exposure for 3 h, the lung wet-to-dry weight ratio which indicated the extent of ALI was significantly increased 6 h after H_2_S exposure, then slightly decreased thereafter (Fig.1).

### 2. Morphology abnormalities induced by H_2_S

**Figure 2:**
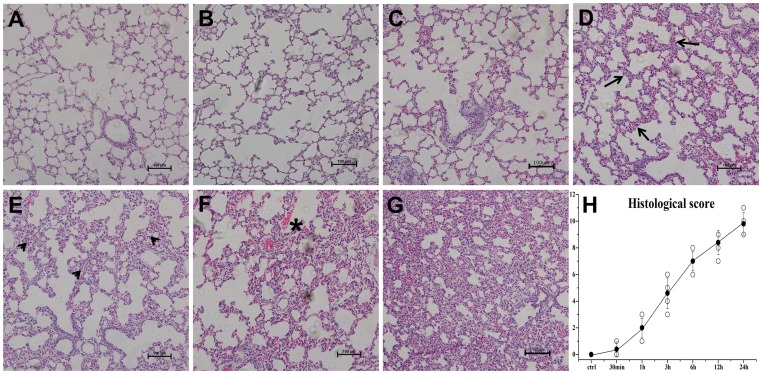
Histopathological changes in lungs after H_2_S exposure at 100 × magnification. A, control group; B, 30 min after H_2_S exposure; C, 1 h after H_2_S exposure; D, 3 h after H_2_S exposure: interlobular septal thickening (arrow); E, 6 h after H_2_S exposure: infiltration of inflammatory cells into interstitium and alveolar spaces (arrowheads); F, 12 h after H_2_S exposure: septal thickening, infiltration of inflammatory cells and haemorrhage (*); G, 24 h after H_2_S exposure: microscopic changes have become much more severe; H, The histological scores. ○: histological scores for all rats; •: error bars: mean±S.E. values for every exposure time point. The histological scores increased in a time-dependent manner.

**Figure 3:**
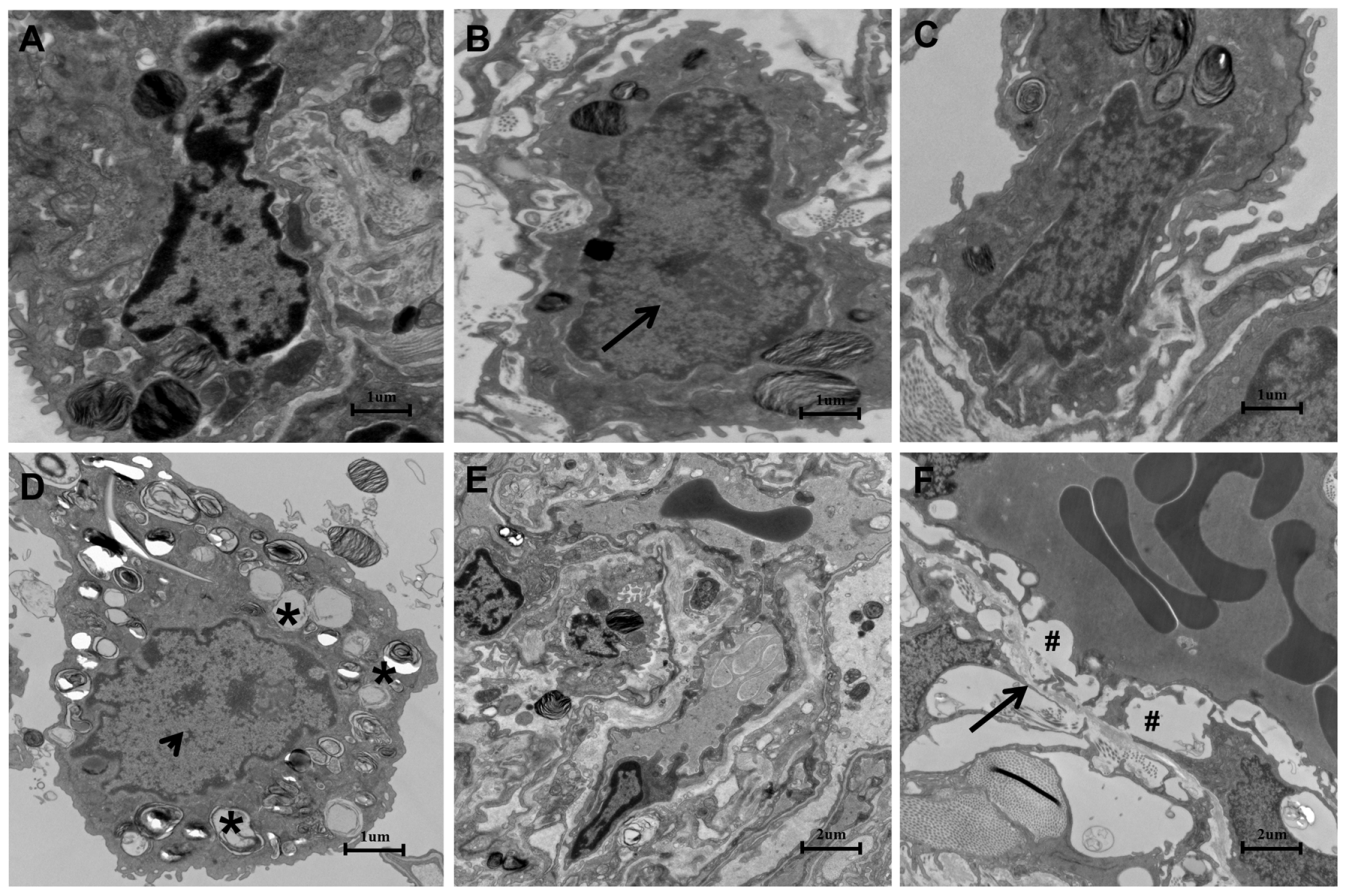
Ultra-structure abnormalities induced by H_2_S in type II alveolar epithelial cells(A,B,C,D Bar  = 1 um) and massive structure of air-blood barrier(E,F Bar  = 2 um). A, control group; B, 6 h after H_2_S exposure: mitochondrial swelling(arrow); C, 12 h after H_2_S exposure, changes between 6 h and 24 h; D, 24 h after H_2_S exposure, depletion of lamellar bodies(*) and mitochondrial shrinking(arrowheads). E, control group, integral blood-air barrier; F, 24 h after H_2_S exposure: segmental blebbing of endothelium(#) and slightly incontinuous of basement membrane(arrow).

To further confirm H_2_S-induced lung injury, the morphological changes were investigated after H_2_S exposure. As depicted in Fig.2, when 3 h, 6 h, 12 h, 24 h after H_2_S exposure (Fig.2D, E, F, G), lung specimens in H_2_S exposed group displayed evident morphological changes, including infiltration of inflammatory cells, hemorrhage and widespread alveolar septum thickening. It was apparent that the extent of the damage gradually increased with time (Fig.2H). However, no obvious pathological changes were observed in 30 min and 1 h group (Fig.2B, C). With the electron microscope assay, the specimens from the H_2_S exposed group displayed evident mitochondrial swelling and shrinking, empty lamellar bodies, nucleus collapse and endothelium rupturing in type II alveolar epithelial cells (Fig.3B, C, D). In addition, ultra-structure abnormalities were also observed in the capillary endothelium and type II alveolar epithelial cells, including capillary hyperemia, segmental blebbing of capillary endothelium and slightly incontinuous of basement membrane(Fig.3F), which manifested the damage effect of H_2_S on the integrity of the blood-air barrier.

### 3. H_2_S induced MMP-2 and MMP-9 expression in rats and A549 cells

**Figure 4:**
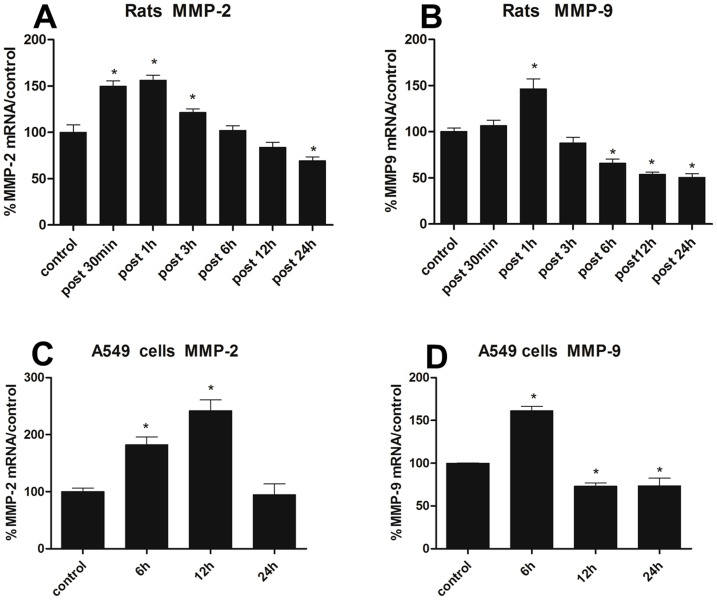
Effects of H_2_S on MMP-2 and MMP-9 mRNA expression. A/B, MMP-2 and MMP-9 mRNA expression in lungs at 30 min, 1 h, 3 h, 6 h, 12 h and 24 h after H_2_S exposure; C/D, MMP-2 and MMP-9 mRNA expression in A549 cells incubated with NaHS(500 μM) for 6 h, 12 h, 24 h. MMP-2 or MMP-9/GAPDH mRNA ratios analyzed by using real-time PCR. Each bar represents the level of MMP-2 and MMP-9 mRNA normalized to the level of GAPDH mRNA, shown as a percentage of the control value. Each data point represents mean±S.E. of mRNA levels from at least three separate experiments in which treatments were performed in triplicates. *Indicates significant difference (p<0.05) when the values were compared to that of the control.

**Figure 5:**
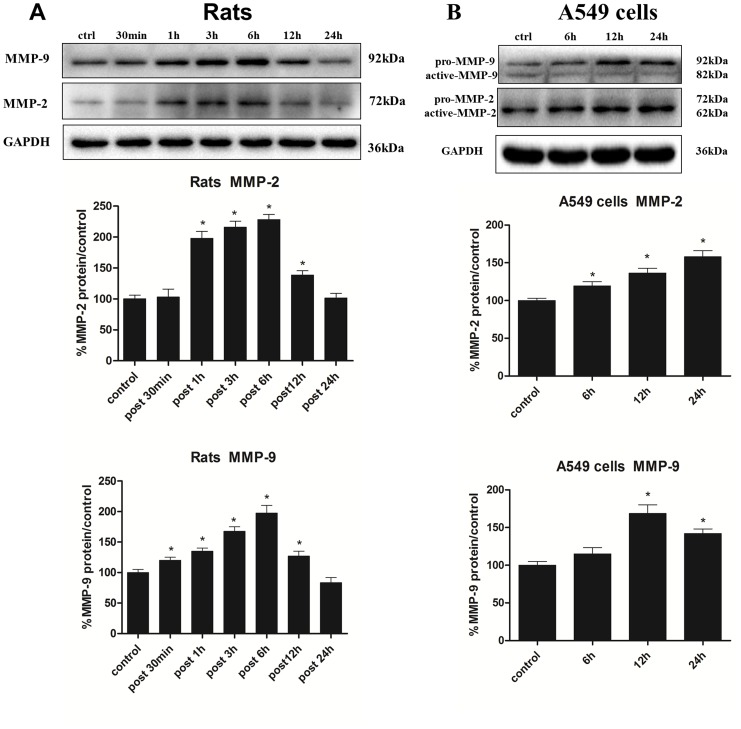
Effects of H_2_S on MMP-2 and MMP-9 protein expression. A, MMP-2 and MMP-9 protein expression in lungs at 30 min, 1 h, 3 h, 6 h, 12 h and 24 h after H_2_S exposure; B, MMP-2 and MMP-9 protein expression in A549 cells incubated with NaHS(500 uM) for 6 h, 12 h and 24 h. Mean values ± S.E. are presented from three independent isolations and three independent samples. *Indicates significant difference (p<0.05) when the values were compared to that of the control.

Having determined the disruption of H_2_S on blood-air barrier, we speculated whether H_2_S exerted its toxic effects through MMP-2 and MMP-9. As shown in Fig.4A and B, MMP-2 and MMP-9 mRNA expression in rats substantially increased 1 h after H_2_S exposure, then gradually decreased and even lower than normal level at 24 h. In vitro study, after the A549 cells incubated with NaHS for 6 h, 12 h and 24 h, as depicted in Fig.4C and D, the MMP-2 and MMP-9 mRNA expression were both significantly increased, and the highest level of MMP-2 mRNA expression was at 12 h, which was approximately 2.4-fold increment when compared with control group, then gradually returned to normal level. The MMP-9 mRNA expression was markedly increased at the time point of 6 h, and then obviously descended. As mRNA level is not always correspond with protein expression, we next evaluated the MMP-2 and MMP-9 expression induced by H_2_S in protein level. As shown in Fig.5A, the protein expression of MMP-2 and MMP-9 in the lung tissues were gradually increased from 1 h to 6 h after H_2_S exposure with a peak of approximately 2-fold increment when compared with the control group at the time point of 6 h, and subsequently decreased to normal level. For the vitro study, the protein expression of MMP-2 in A549 cells was gradually increased when incubated with NaHS for 6 h, 12 h and 24 h, and the highest level was 1.6-fold increment when compared with the control group at 24 h. For MMP-9, the protein expression was markedly increased in all time points induced by H_2_S, and with a 1.7-fold increment at the time point of 12 h (Fig.5B).

### 4. Doxycycline ameliorated H_2_S-induced ALI by the inhibition of MMP-2 and MMP-9

**Figure 6:**
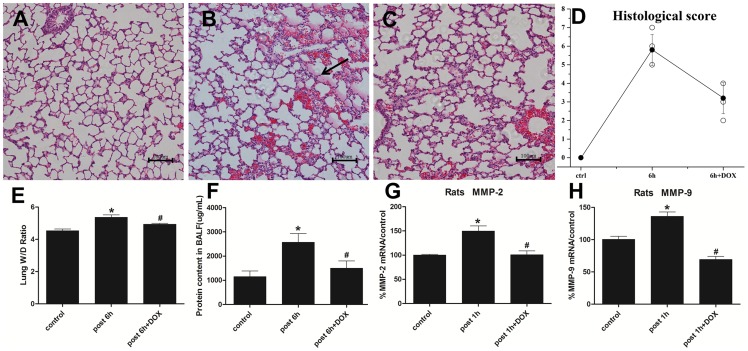
DOX ameliorated H_2_S-induced ALI and inhibited MMP-2 and MMP-9 mRNA expression. A, control group; B, 6 h after H_2_S exposure: alveolar edema fluid (arrow); C, DOX + H_2_S post 6 h group; D, The histological scores, ○: histological scores for all rats; •: error bars: mean±S.E. values for each group. E, wet/dry ratio; F, protein content in BALF. G/H, rats were pre-treated with DOX(20 mg/kg/day) for consecutive 7 days, then MMP-2 and MMP-9 mRNA expression were investigated at the time point of 1 h after H_2_S exposure. MMP-2 or MMP-9/GAPDH mRNA ratios were analyzed by using real-time PCR. Each bar represents the levels of MMP-2 and MMP-9 mRNA normalized to the level of GAPDH mRNA, shown as a percentage of the control value. Mean values ± S.E. are presented from three independent experiments. * Indicates significant difference (p<0.05) versus control group. # Indicates significant difference (p<0.05) versus H_2_S post 1 h or 6 h group.

To determine whether MMP-2 and MMP-9 up-regulation played a critical role in H_2_S -induced ALI, doxycycline(DOX), the most potent nonspecific MMP inhibitor with the effects of degrading pro-MMP zymogen and inhibiting MMP mRNAs transcription [44], [45], was used in the present work. From the perspective of lung injury, as ALI/ARDS is characterized by alveolar destruction, lung edema and protein leakage from impaired blood-air barrier into the BALF where may exist a higher content of MMP-2 and MMP-9 [46]. As we speculated, DOX significantly decreased the alveolar edema fluid (Fig.6B, C), which was supported the result of wet-to-dry weight ratio (Fig.6E). And the elevated average BALF protein content in H_2_S exposed group was also obviously reduced in DOX + H_2_S treated group (Fig.6F).

**Figure 7:**
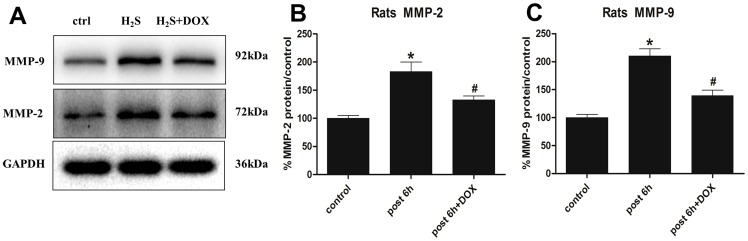
Effects of DOX on MMP-2 and MMP-9 protein expression. Rats were pre-treated with DOX(20 mg/kg/day) for consecutive 7 days, then MMP-2 and MMP-9 protein expression were investigated 6 h after H_2_S exposure. Mean values ± S.E. are presented from three independent isolations and three independent samples. *Indicates significant difference (p<0.05) versus control group. # Indicates significant difference (p<0.05) versus H_2_S post 6 h group.

**Figure 8:**
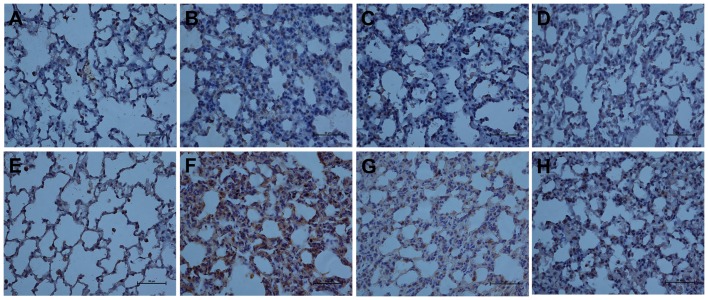
The immunohistochemical expression of MMP-2 and MMP-9 at 400× magnification. A/E, control group for MMP-2 and MMP-9; B/F, 6 h after H_2_S exposure for MMP-2 and MMP-9; C/G, DOX + H_2_S post 6 h group for MMP-2 and MMP-9; D/H, DXM + H_2_S post 6 h group for MMP-2 and MMP-9. Pre-treatment with DOX or DXM reduced the up-regulated immunohistochemical expression of MMP-2 and MMP-9 after H_2_S exposure.

In addition, DOX remarkably decreased MMP-2 and MMP-9 mRNA expression by 33% and 49% respectively when compared with H_2_S exposed group (Fig.6G, H). Similarily, the protein level of MMP-2 and MMP-9 in lung tissues was obviously descended in DOX + H_2_S treated group than in H_2_S solo exposed group(Fig.7A, B, C). For immunohistochemical assay, MMP-2 and MMP-9 were both strongly expressed in the injured lung tissues of H_2_S exposed rats, however, the expression was attenuated by DOX (Fig.8B, C, F, G).

### 5. Dexamethasone attenuated H_2_S-induced ALI in rats

**Figure 9:**
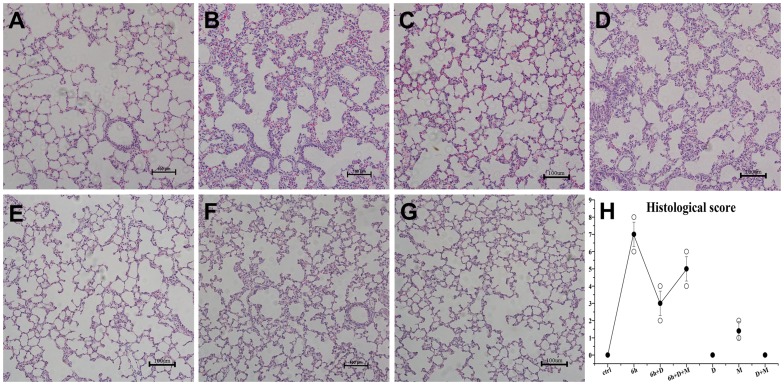
DXM attenuated H_2_S-induced ALI in rats at 100× magnification. A, control group; B, 6 h after H_2_S exposure; C, DXM + H_2_S post 6 h group; D, DXM and MIF + H_2_S post 6 h group; E, DXM solo treated group; F, MIF solo treated group; G, DXM and MIF treated unexposed group; H, The histological scores, ○: histological scores for all rats; •: error bars: mean±S.E. values for each group. DXM significantly attenuated H_2_S-induced ALI, and MIF partly blocked the effect.

**Figure 10:**
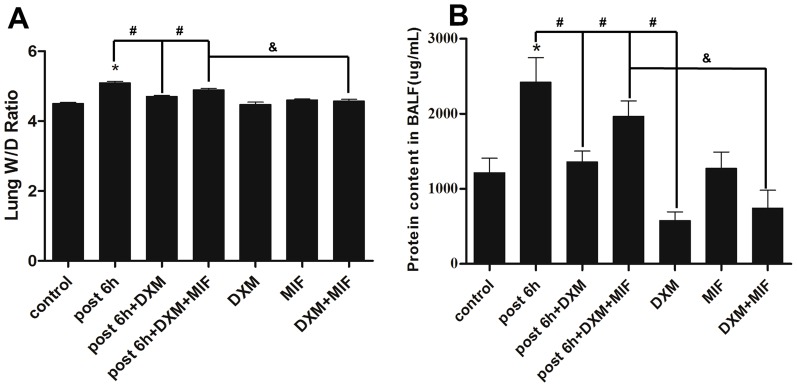
Wet/dry ratio and protein content in BALF. A, wet/dry ratio; B, protein content in BALF. Mean values ± S.E. are presented from three independent experiments. * Indicates significant difference (p<0.05) versus control group. # Indicates significant difference (p<0.05) versus DXM + H_2_S post 6 h group; & Indicates significant difference (p<0.05) between H_2_S + DXM + MIF and DXM + MIF treated group.

The evident histopathologic abnormalities of lung specimens in H_2_S exposed group have been investigated aforementioned. However, these processes can be partially retarded by DXM(Fig.9B,C). Moreover, MIF, the GR antagonist, obviously blocked the protective effects of DXM (Fig.9D). Meanwhile, DXM significantly attenuated H_2_S-induced lung edema by decreasing the wet-to-dry weight ratio (Fig.10A). As shown in Fig.10B, the average protein content in BALF from H_2_S exposed group increased 2-fold than control group at the time point of 6 h after H_2_S exposure, suggesting massive protein leakage due to increased pulmonary permeability. However, when H_2_S exposed rats were pre-treated with DXM, average BALF protein content was significantly reduced and the effect could be partially blocked by MIF. In addition, the BALF protein content from DXM solo treated group was a half of normal level.

### 6. Dexamethasone alleviated MMP-2 and MMP-9 expression

**Figure 11:**
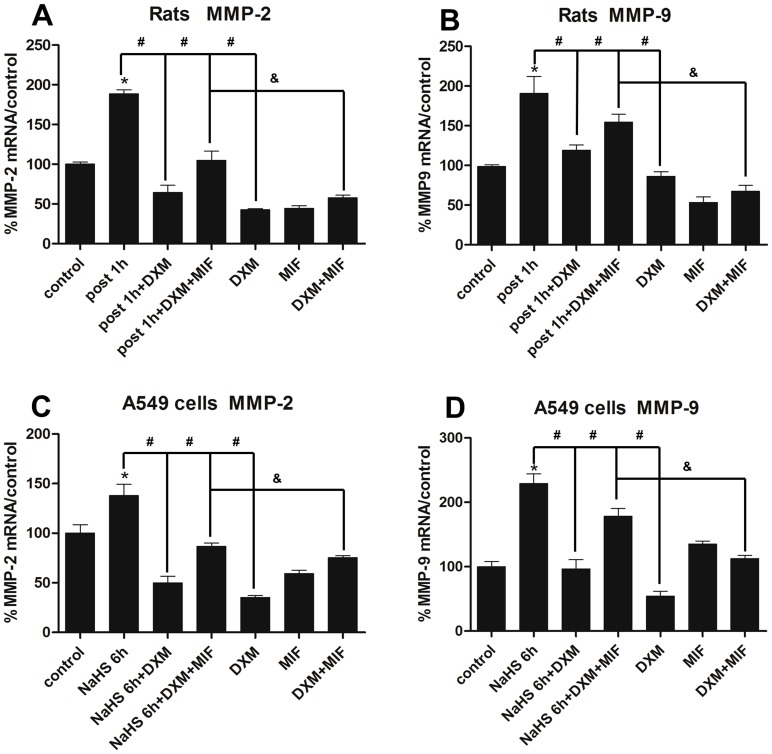
Effects of DXM on MMP-2 and MMP-9 mRNA expression. A/B, rats were pre-treated with DXM(2 mg/kg/day) and/or MIF(10 mg/kg/q12 h) for consecutive 3 days, then MMP-2 and MMP-9 mRNA expression were investigated 1 h after H_2_S exposure. C/D, A549 cells were pre-treated with DXM(100 nM) and/or MIF(1 μM) for 24 h, then incubated with NaHS(500 μM) for 6 h to investigate MMP-2 and MMP-9 mRNA expression. MMP-2 or MMP-9/GAPDH mRNA ratios analyzed by using real-time PCR. Each bar represents the levels of MMP-2 and MMP-9 mRNA normalized to the level of GAPDH mRNA, shown as a percentage of the control value. Each data point represents mean±S.E. of mRNA levels from at least three separate experiments in which treatments were performed in triplicates. *Indicates significant difference (p<0.05) versus control group. # Indicates significant difference (p<0.05) versus DXM + H_2_S/NaHS exposed group; & Indicates significant difference (p<0.05) between H_2_S/NaHS + DXM + MIF and DXM + MIF treated group.

**Figure 12:**
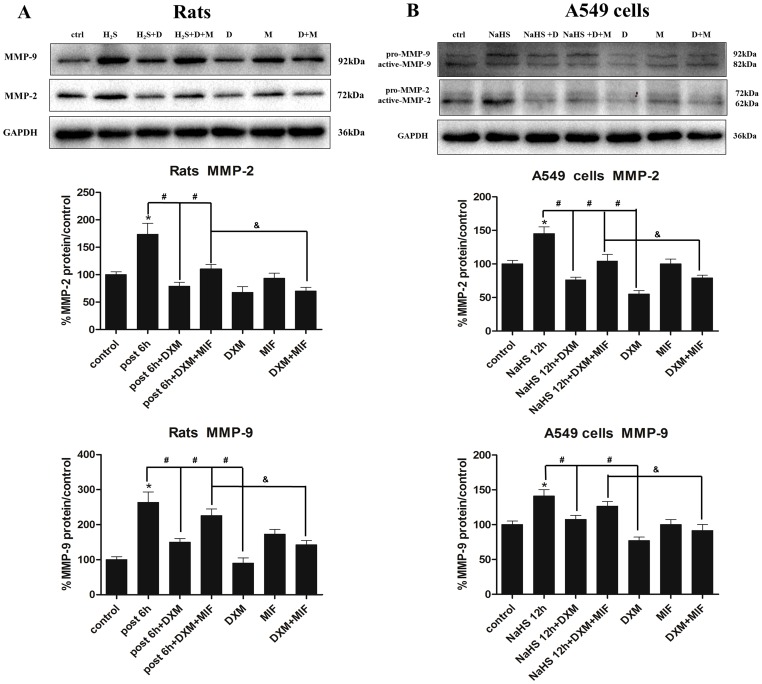
Effects of dexamethasone on MMP-2 and MMP-9 protein expression. A, rats pret-reated with DXM(2 mg/kg/day) and/or MIF(10 mg/kg/q12 h) for consecutive 3 days, then investigate MMP-2 and MMP-9 protein expression were investigated at the time point of 6 h after H_2_S exposure. B, A549 cells were pre-treated with DXM(100 nM) and/or MIF(1 μM) for 24 h, then incubated with NaHS(500 μM) for 12 h and MMP-2 and MMP-9 protein expression were detected. Mean values ± S.E. are presented from three independent isolations and three independent samples. *Indicates significant difference (p<0.05) versus control group. # Indicates significant difference (p<0.05) versus DXM + H_2_S/NaHS exposed group; & Indicates significant difference (p<0.05) between H_2_S/NaHS + DXM + MIF and DXM + MIF treated group.

Then we questioned whether up-regulation of GR by DXM was associated with the suppression of MMP-2 and MMP-9. As the highest level for mRNA and protein expression of MMP-2 and MMP-9 in H_2_S exposed lung tissues were at 1 h and 6 h respectively aforementioned, and thus the exposed rats were killed 1 h and 6 h afterward to detect mRNA and protein expression. In rat model, as depicted in Fig.11A and B, DXM substantially decreased MMP-2 and MMP-9 mRNA expression by 65% and 37% respectively when compared with H_2_S exposed group, and MMP-2 expression was even lower than control. Moreover, MIF partially retarded the effect of DXM in both mRNA and protein level, though MIF up-regulated MMP-9 protein expression in MIF solo treated group (Fig.12A). In addition, as shown in Fig.8B, D, F and H, DXM pre-treatment retarded the elevated immunohistochemical expression of MMP-2 and MMP-9 after H_2_S exposure.

In vitro study, according to the time course of MMP-2 and MMP-9 expression in A549 cells incubated with NaHS(Fig.4C,D; Fig.5B), we evaluated the effect of DXM on MMP-2 and MMP-9 mRNA expression at the time point of 6 h as well as the protein expression at 12 h in parallel with the rats model. As shown in Fig.11C and D, DXM substantially decreased MMP-2 and MMP-9 mRNA expression by 64% and 58% when compared with NaHS treated group, and this effect could be restrained by co-treatment with MIF. In addition, MMP-2 and MMP-9 mRNA expression were suppressed by 65% and 45% respectively in DXM solo treated group. While MIF slightly increased MMP-9 mRNA expression, and the MMP-2 mRNA expression was antipodal in MIF solo treated group. In protein level, DXM obviously alleviated MMP-2 and MMP-9 protein expression in the present or absent of NaHS, and MIF was able to retarded the effect of DXM, which was in line with the mRNA expression (Fig.12B).

## Discussion

H_2_S, as a highly toxic gas, could inhibit the cytochrome oxidase system, with a permissible exposure limit only can be up to 50 ppm for a single period up to 10 min [47]. Human exposure to H_2_S poses an immediate health and life hazard, and the death was closely related to H_2_S-induced severe lung injury [48], [49]. The MMP-2 and MMP-9 are believed to be responsible for the destruction of blood-air barrier by degrading ECM during the pathogenesis of ALI/ARDS [50].

In the present study, pathological changes from H_2_S exposed lung tissues displayed aggravation of diffuse alveolar damage such as inflammatory cells infiltration and alveolar septum thickening, in addition, some ultra-structure abnormalities were also observed, including type II alveolar epithelial cells apoptosis, which was consistent with other study that alveolar epithelium displayed ultrastructural alterations after H_2_S exposure [51]. The protein content in BAFL was obviously increased after H_2_S exposure, implying the leakage of proteins into BALF from the impaired blood-air barrier. Moreover, the pulmonary edema evaluated by wet/dry weight ratio was markedly increased after H_2_S exposure as well. These results, taken together, providing the definite evidence for H_2_S-induced ALI and were involved in the formation of scathing manifestations in lung CT images after H_2_S exposure, such as patchy shadow and pleural effusion [49].

The crucial roles of MMPs in the development of ALI has been demonstrated in the MMPs knockout mouse [17], [18]. In the present work, for the first time, we indicated that H_2_S significantly increased MMP-2 and MMP-9 expression at mRNA and protein level in both vivo and vitro study, which prompted the critical contributions of MMP-2 and MMP-9 to the development of H_2_S-induced ALI. Supporting our results, recent studies demonstrated that the increased expression of MMP-2 and MMP-9 were also observed in the exposure of other hazard gases, like phosgene and chlorine [22], [52]. To prove the critical roles of MMP-2 and MMP-9 in H_2_S-induced ALI, we indicated that the MMP inhibitor DOX obviously attenuated H_2_S-induced ALI via the direct suppression of MMP-2 and MMP-9, which was consistent with other studies that DOX inhibited the mRNA and protein expression of MMP-2 and MMP-9, and attenuated the symptoms of ALI including aggravating alveolar destruction, neutrophil migration to the airspaces of lung and protein leakage in BAFL [33], [34], [53], [54].

DXM, a powerful and widely used glucocorticoid, was reported to exert protective effects in various pulmonary conditions. It was previously indicated that DXM could up-regulate GR to mediate the suppression of MMP-2 and MMP-9 in the absence of specific MMP inhibitors [20]–[25]. Therefore, in the present work, we speculated whether DXM protected against H_2_S-induced ALI and whether this effect was through the inhibition of MMP-2 and MMP-9. Our results revealed that DXM significantly attenuated H_2_S-induced ALI in rats including ameliorative pathologic changes, decrement of wet/dry weight ratio and the protein content in BALF. It might be attribute to that DXM attenuated the H_2_S mediated up-regulation of MMP-2 and MMP-9 expression, and perhaps a direct effect on the ECM to maintain the integrity of blood-air barrier, which was manifested in phosgene and lipopolysaccharide induced ALI [22], [55]. Moreover, the protective effects of DXM could partially blocked by MIF, the GR antagonist, which further proved that GR was also involved in the pathogenesis of ALI/ARDS [20], [21] and the inhibitory effects of DXM on MMP-2 and MMP-9 expression [22]–[25].

In summary, we present for the first time that H_2_S increases MMP-2 and MMP-9 expression which might aggravate the development of ALI. DXM exerts protective effects by attenuating MMP-2 and MMP-9 expression through the up-regulation of GR. Therefore, MMP-2 and MMP-9 might represent novel pharmacological targets for the treatment of ALI induced by H_2_S and other hazard gases, and further research will focus on the mechanisms of MMP-2 and MMP-9 down-regulation mediated by DXM.
